# A Global Genomic Characterization of Nairoviruses Identifies Nine Discrete Genogroups with Distinctive Structural Characteristics and Host-Vector Associations

**DOI:** 10.4269/ajtmh.15-0917

**Published:** 2016-05-04

**Authors:** Peter J. Walker, Steven G. Widen, Thomas G. Wood, Hilda Guzman, Robert B. Tesh, Nikolaos Vasilakis

**Affiliations:** CSIRO Health and Biosecurity, Australian Animal Health Laboratory, Geelong, Victoria, Australia; Department of Biochemistry and Molecular Biology, The University of Texas Medical Branch, Galveston, Texas; Center for Biodefense and Emerging Infectious Diseases and Department of Pathology, Center for Tropical Diseases, and Institute for Human Infections and Immunity, The University of Texas Medical Branch, Galveston Texas

## Abstract

Nairoviruses are primarily tick-borne bunyaviruses, some of which are known to cause mild-to-severe febrile illness in humans or livestock. We describe the genome sequences of 11 poorly characterized nairoviruses that have ecological associations with either birds (Farallon, Punta Salinas, Sapphire II, Zirqa, Avalon, Clo Mor, Taggert, and Abu Hammad viruses), rodents (Qalyub and Bandia viruses), or camels (Dera Ghazi Khan virus). Global phylogenetic analyses of proteins encoded in the L, M, and S RNA segments of these and 20 other available nairovirus genomes identified nine well-supported genogroups (Nairobi sheep disease, Thiafora, Sakhalin, Keterah, Qalyub, Kasokero, Dera Ghazi Khan, Hughes, and Tamdy). Genogroup-specific structural variations were evident, particularly in the M segment encoding a polyprotein from which virion envelope glycoproteins (Gn and Gc) are generated by proteolytic processing. Structural variations include the extension, abbreviation, or absence sequences encoding an O-glycosylated mucin-like protein in the N-terminal domain, distinctive patterns of conserved cysteine residues in the GP38-like domain, insertion of sequences encoding a double-membrane-spanning protein (NSm) between the Gn and Gc domains, and the presence of an alternative long open reading frame encoding a viroporin-like transmembrane protein (Gx). We also observed strong genogroup-specific associations with categories of hosts and tick vectors.

## Introduction

Nairoviruses are arthropod-borne bunyaviruses (genus *Nairovirus*, family *Bunyaviridae*). Like other bunyaviruses, nairoviruses are enveloped, negative-sense, single-stranded RNA viruses containing three segments (L, M, and S) of genomic RNA. They are transmitted primarily by ticks (Ixodidae and Argasidae) and naturally infect birds, small mammals (bats, rodents, lagomorphs, moles, shrews, and hedgehogs), and ungulates.^1^ In some cases, nairovirus infections spillover to humans, causing illnesses that can range from headache or mild fever to fatal hemorrhagic fever.^2^–^5^ From a public health perspective, Crimean-Congo hemorrhagic fever virus (CCHFV) is considered to be the most significant nairovirus. Over 5,000 human cases of CCHF have been reported since the disease was first recognized in the Crimea in 1944, with case-fatality rates of up to 30% and a geographic distribution that extends through Africa, the Middle East, Eastern Europe, and Asia.^1^,^6^,^7^ Nairobi sheep disease virus (NSDV) is a nairovirus of veterinary importance, causing hemorrhagic gastroenteritis in sheep and goats in Africa and Asia with mortality rates of up to 90%.^8^,^9^ More than 50 other nairoviruses have been isolated from ticks or vertebrates, many of which are poorly characterized. Of these, 35 viruses are currently assigned to seven serogroups (CCHF, Nairobi sheep disease, Hughes, Dera Ghazi Khan [DGK], Qalyub, Sakhalin, and Thiafora)^1^ and recent evidence indicates that seven other viruses should be assigned to two additional serogroups (Kasokero and Keterah).^10^ All viruses falling within a serogroup are currently assigned to the same virus species.^11^ Several nairo-like virus genome sequences have also been detected in ticks, other arachnids (spiders), and an insect (water strider), but the biological significance of these findings is currently unclear.^12^,^13^

Here, we report the genome sequences of 11 poorly described nairoviruses from the Hughes, DGK, Qalyub, and Sakhalin serogroups. With this greatly enhanced genomic resource, we conduct a global analysis of all 31 available nairovirus genome sequences and identify nine genogroups of tick-associated nairoviruses that are well supported phylogenetically, comprise viruses encoding proteins with common structural features and have strong associations with categories of hosts and vectors.

## Materials and Methods

### Description of viruses.

Bandia virus (BDAV) was isolated from a rodent (*Mastomys* sp.) and subsequently from soft ticks (*Ornithodorus erraticus*) collected from rodent burrows in Senegal; Qalyub virus (QYBV) was isolated from soft ticks (*O. erraticus*) collected from a rodent nest in Egypt.^14^,^15^ BDAV and QYBV cross-react in complement-fixation (CF) tests and have been assigned to the Qalyub serogroup.^16^ Farallon virus (FARV) from California, Punta Salinas virus (PSV) from Peru, Sapphire II virus (SAPV) from Texas, and Zirqa virus (ZIRV) from Abu Dhabi were each isolated from soft ticks (*Ornithodorus* spp. or *Argas cooleyi*) collected from birds or bird nests.^17^–^20^ They cross-react strongly by indirect immunofluorescence with Hughes virus and have been assigned to the Hughes serogroup.^21^ Avalon virus (AVAV) from Newfoundland, Clo Mor virus (CMV) from Scotland, and Taggert virus (TAGV) from Macquarie Island in the Southern Ocean were each isolated from hard ticks (*Ixodes uriae*) collected from seabirds or seabird nests.^22^,^23^ They are related antigenically to SAKV and have been assigned to the Sakhalin serogroup. DGKV from Pakistan was isolated from hard ticks (*Hyalomma* spp.) collected from camels^24^ and Abu Hammad virus (AHV) was isolated from soft ticks (*Argas hermanni*) collected from a pigeon house in Egypt.^25^ DGKV and AHV have been assigned to the DGK serogroup.^26^ GenBank accession numbers for genome sequences or partial L gene sequences of these and other nairoviruses used in this study are provided in Table 1.

### Growth and passage history.

The 11 nairoviruses sequenced in this study were obtained from the World Reference Center for Emerging Viruses and Arboviruses at the University of Texas Medical Branch, Galveston, TX, and from the Centers for Disease Control and Prevention, Fort Collins, CO. Each of the viruses was initially isolated by intracranial inoculation of newborn mice and has been maintained by serial passage in suckling mice (SM). Stains and passage histories of viruses sequenced were as follows: BDAV strain IPD-A611 (SM9), QYBV strain ErAg 370 (SM4), FARV strain CalAr 846 (SM7), PSV strain CalAr 888 (SM8), SAPV strain RML 52301-14 (SM6), ZIRV strain A2070-1 (SM11), AVAV strain CanAr 173 (SM10), CMV strain ScotAr 7 (SM10), TAGV strain Aus MI14850 (SM8), DGKV strain JD254 (SM11), and AHV strain EgArt 1194 (SM8).

### Extraction of viral RNA.

For all viruses supernatant fluid from a culture of infected Vero cells was used for RNA extraction and sequencing. Supernatants were harvested and clarified by low-speed centrifugation (2,000 × *g*, 10 minutes at 4°C) once cytopathic effect was advanced. One millilitre of clarified supernatant from each virus was treated with a cocktail of DNases (14 U Turbo DNase [Ambion, Austin, TX], 20 U Benzonase [EMD Millipore, Billerica, MA], and 20 U RNase One [Promega, Madison, WI]) for 1 hour at 37°C. Viral RNA was then extracted using Trizol and resuspended in 50 μL RNase/DNase and protease-free water (Ambion).

### Next generation sequencing.

Viral RNA (∼0.9 μg) was fragmented by incubation at 94°C for 8 minutes in 19.5 μL of fragmentation buffer (Illumina 15016648). A sequencing library was prepared from the RNA sample using an Illumina TruSeq RNA v2 kit (Illumina, San Diego, CA) following the manufacturer's protocol. The sample was sequenced on an Illumina HiSeq 1500 using the 2 × 50 paired-end protocol. Reads in FASTQ format were quality filtered, and any adapter sequences were removed, using Trimmomatic^27^ software (Institute of Botany and Molecular Genetics, AAchen University). The de novo assembly program ABySS^28^ (Michael Smith Genome Sciences Center, Vancouver, Canada) was used to assemble the reads into contigs, using several different sets of reads, and *k* values from 20 to 40. In several samples (CMV, FARV, PSV, TFAV, and ZIRV) host reads were filtered out before de novo assembly. The longest contigs were selected and reads were mapped back to the contigs using bowtie2^29^ and visualized with the Integrated Genomics Viewer^30^ (Broad Institute, Boston, MA) to verify that the assembled contigs were correct. A total of 10.3, 14.1, 10.7, 47.7, 11.9, 17.1, 14.3, 14.9, 10.4, 11.0, and 14.9 million reads were generated for the samples containing AHV, AVAV, BDAV, CMV, DGKV, FARV, PSV, QYBV, SAPV, TAGV, and ZIRV, respectively. Reads mapping to the virus in each sample comprised ∼64,000 (0.6%), ∼256,000 (1.8%), ∼63,300 (0.7%), ∼4,000 (0.01%), ∼165,600 (1.4%), ∼85,000 (0.5%), ∼50,000 (0.4%), ∼402,000 (2.7%), ∼143,000 (1.4%), ∼7,800 (0.07%), and ∼84,000 (0.6%), respectively.

### Phylogenetic analysis.

Amino acid (aa) alignments were created using ClustalW in MEGA 6.0^31^ (Center for Evolutionary Medicine and Informatics, The Biodesign Institute, Tempe, AZ) and ambiguously aligned regions removed using the Gblocks program with default parameters.^32^ The resulting sequence alignments were used to infer maximum-likelihood (ML) phylogenetic trees in MEGA6.0, employing the WAG model of aa substitution, and either nearest-neighbor interchange (NNI) and subtree pruning and regrafting branch swapping. The phylogenetic robustness of each node was determined using 1,000 bootstrap replicates and NNI branch swapping.

#### Bioinformatic analysis.

Analysis of the structural characteristics of nairovirus proteins was conducted using bioinformatics resources accessed through the ExPASy portal (http://www.expasy.org/proteomics) including Translate (nucleotide sequence translation), TMHMM (transmembrane protein prediction), SignalP (signal peptidase cleavage sites), NetPhos (phosphorylation sites), NetOGlyc (O-linked glycosylation sites), and NetNGlyc (N-linked glycosylation sites).

## Results

### Genome sequences.

Nucleotide sequences of the complete coding regions of the L, M, and S segments were obtained for BDAV, QYBV, FARV, SAPV, PSV, ZIRV, AVAV, CMV, DGKV, AHV, and TAGV. Translation of the nucleotide sequences confirmed that each L segment contained a single long open reading frame (ORF) encoding the nairovirus multifunctional RNA-dependent RNA polymerase (L protein). The M segments encoded multiple-membrane spanning polyglycoproteins that included domains corresponding to the nairovirus envelope glycoproteins (Gn and Gc proteins). The S segments contained single long ORFs encoding the nairovirus nucleoproteins (N proteins). We also included in our data set the genome sequences of 13 nairoviruses that were already available in GenBank and seven complete nairovirus-like genome sequences reported from metagenomic analyses of ticks, spiders, or insects.^12^,^13^ Although the complete genome sequences of several other nairoviruses (Chim, Caspiy, Tamdy, Burana, Geran, Sakhalin, and Paramushir viruses) have been reported previously (in Russian),^33^–^38^ the viruses have not been deposited in a public database and were not available for this study. GenBank accession numbers and details of the sources of isolation/detection of all the viruses used in the study are provided in Table 1.

### Genogroup assignments.

**Figure 1.**
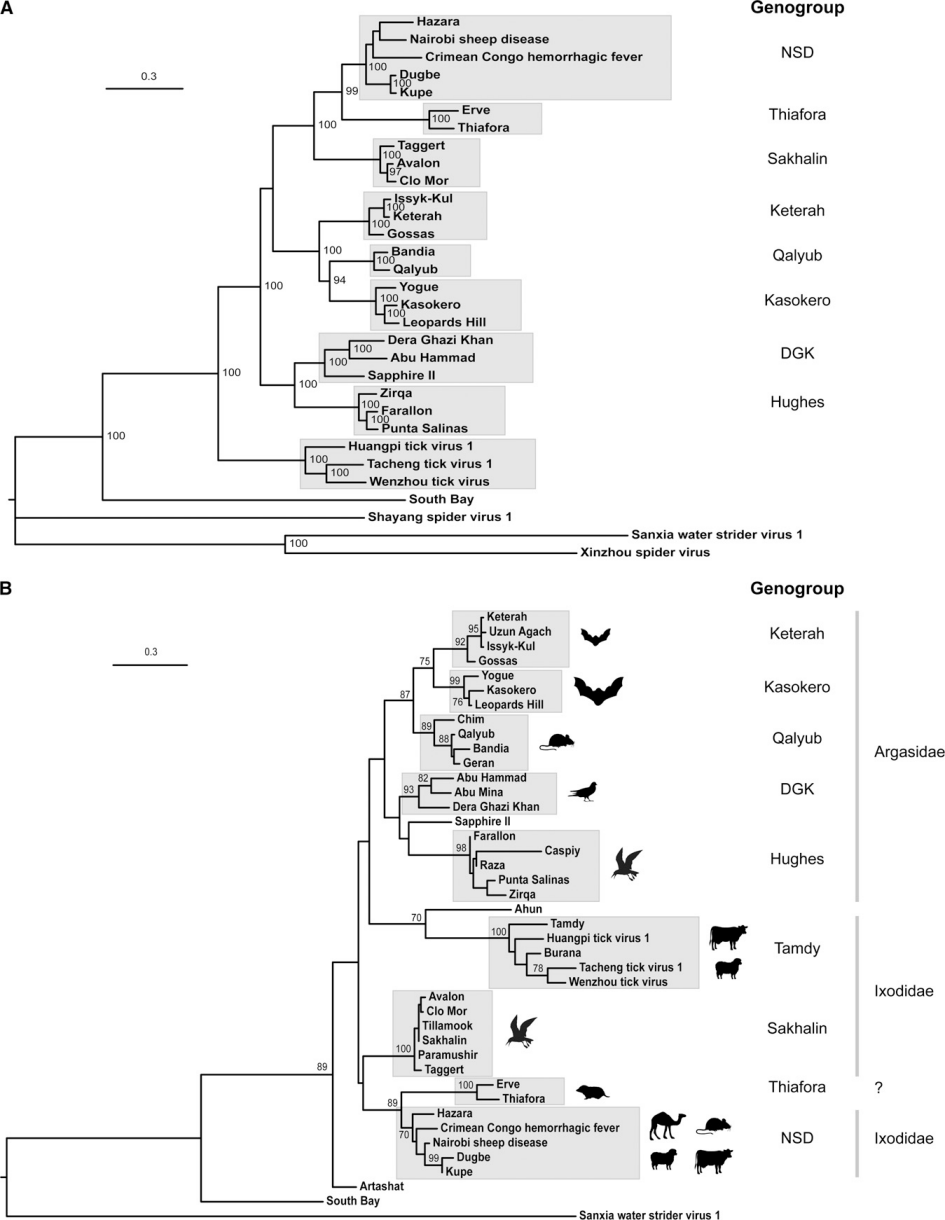
Maximum-likelihood phylogenetic trees inferred from Clustal X alignments of amino acid sequences of proteins encoded in nairovirus L RNA segments. (**A**) Tree inferred from full-length L protein sequences. (**B**) Tree inferred from partial sequence (154 amino acids) of L proteins indicating the sources of isolation of the viruses from vertebrate hosts and hard or soft tick vectors. Genogroup assignments of clades in each tree are shown. The description of virus isolates and GenBank accession numbers of sequences used in the alignments are provided in Table 1. Horizontal branch lengths are drawn to a scale of amino acid substitutions/site and all bootstrap proportion values ≥ 70% are shown.

Initially, we inferred a ML phylogenetic tree using the complete L protein sequences of the 31 nairoviruses in our data set. The tree included nine robustly supported clades (bootstrap proportion [BSP] ≥ 98%) comprising 27 viruses that appeared to be primarily tick associated and we assigned these as genogroups (Figure 1A

). The Thiafora (TFAV and Erve virus [ERVV]), Sakhalin (TAGV, AVAV, and CMV), Keterah (Issyk-Kul virus [IKV], Keterah virus [KTRV], and Gossas virus [GOSV]), Qalyub (BDAV and QYBV), Kasokero (Yogue virus [YOGV], Kasokero virus [KKOV], and Leopards Hill virus [LPHV] and Hughes (ZIRV, FARV, and PSV) genogroups comprised viruses that had previously been assigned to the corresponding serogroups.^1^ However, SAPV, which has been assigned to the Hughes serogroup clearly clustered with DGKV and AHV, and so was assigned to the DGK genogroup. Furthermore, viruses that have previously been assigned to either the Nairobi sheep disease serogroup (NSDV, Dugbe virus [DUGV], and Kupe virus [KUPV]) or the Crimean Congo hemorrhagic fever serogroup (CCHFV and Hazara virus [HAZV]) formed a single clade that we assigned as the NSD genogroup. The ninth genogroup comprised three viral sequences (Huangpi tick virus 1 [HTV1], Tacheng tick virus 1 [TTV1], and Wenzhou tick virus [WTV]) that were obtained by metagenomic analysis of ticks from China and for which virus isolates are not currently available. South Bay virus (SBV) (also isolated from ticks) was more deeply rooted in the tree. Nairo-like viral sequences obtained from spiders and an insect formed the most deeply rooted branches of the tree.

To extend the analysis of genogroups, we then constructed an ML tree from an alignment of sequences of a short region of the L protein that was available for 42 nairoviruses (Figure 1B). The region comprised 466 nucleotides or 154 aa extending from a region upstream of conserved premotif A to the center of conserved motif A.^39^ Although the tree was less robustly supported at the intermediate and deeper nodes, there was robust support (BSP ≥ 89%) for the assignment of additional viruses to the nine genogroups identified in Figure 1A. These included Uzun Agach virus (UZV) (Keterah genogroup); Chim virus (CHIMV), and Geran virus (GERV) (Qalyub genogroup); Abu Mina virus (AMV) (DGK genogroup); Caspiy virus (CASV) and Raza virus (RAZV) (Hughes genogroup); and Tillamook virus (TILV), Sakhalin virus (SAKV) and Paramushir virus (PMRV) (Sakhalin genogroup). There was also robust bootstrap support (BSP = 100%) for a clade comprising Tamdy virus (TDYV) and Burana virus (BURV) as well as the sequences obtained from ticks in China (HTV1, TTV1, and WTV); this was therefore assigned as the Tamdy genogroup. The assignment of SAPV and Ahun virus to genogroups was not robustly supported in this tree. Like SBV, Artashat virus (also isolated from ticks) was deeply rooted in the tree.

### Evidence of genome segment reassortment.

**Figure 2.**
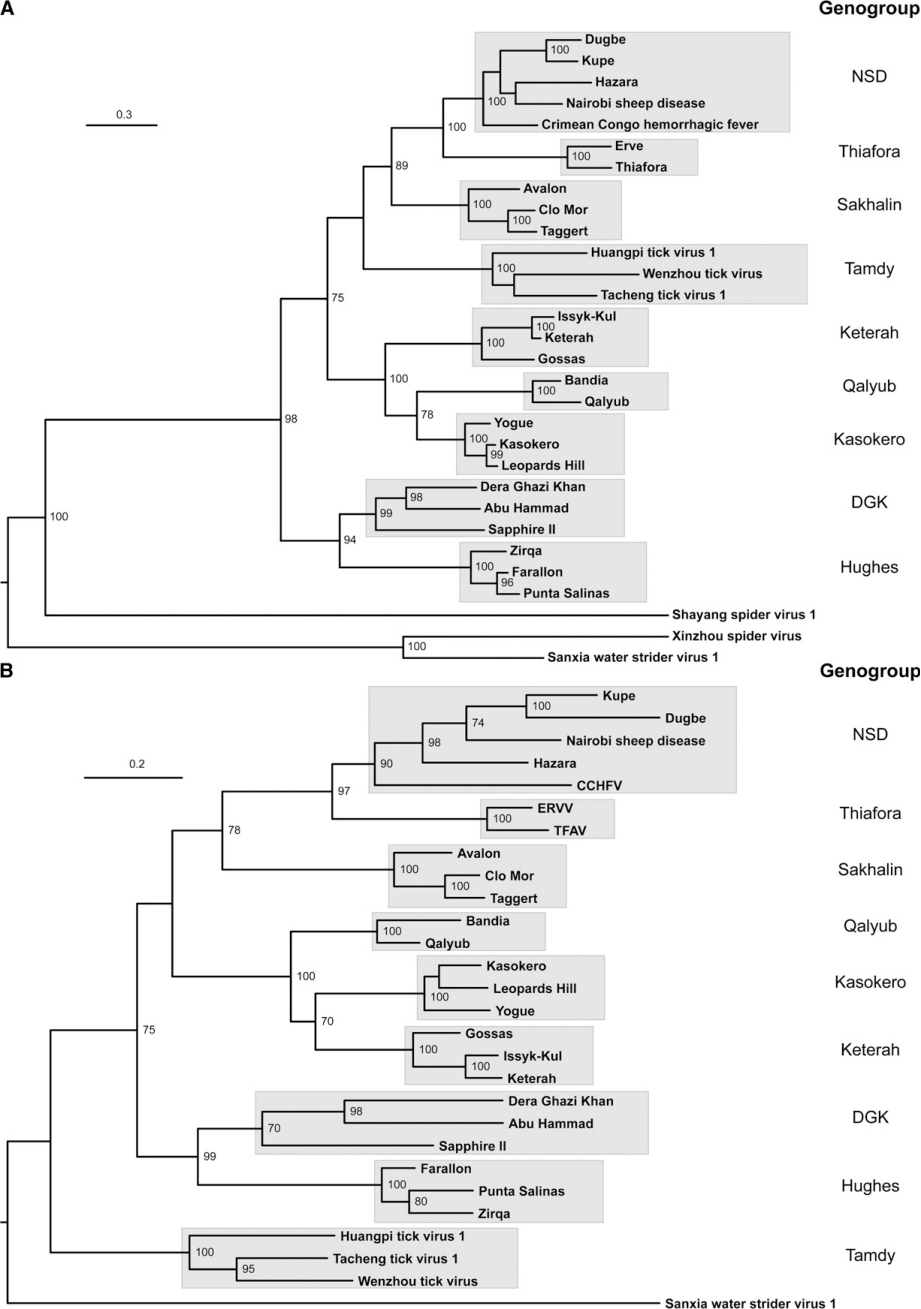
Maximum-likelihood phylogenetic trees inferred from Clustal X alignments of amino acid sequences of proteins encoded in nairovirus S and M RNA segments. (**A**) Tree inferred from full-length N protein sequences. (**B**) Tree inferred from concatemerized sequences of full-length Gn and Gc proteins. Genogroup assignments of clades in each tree are shown. The description of virus isolates and GenBank accession numbers of sequences used in the alignments are provided in Table 1. Horizontal branch lengths are drawn to a scale of amino acid substitutions/site and all bootstrap proportion values (BSP) ≥ 70% are shown.

To assess evidence of genome segment reassortment, we generated ML phylogenetic trees using the sequences of proteins encoded in the S and M genome segments. The S segment tree was inferred from an alignment of complete N protein aa sequences (Figure 2A

); the M segment tree was inferred from concatenated aa sequences of the complete Gn and Gc coding regions (Figure 2B). Although some of the deeper nodes were not well supported, each tree displayed the same nine well-supported clades (BSP ≥ 75%) identified from the L protein tree (Figure 1A), confirming the genogroup assignments and indicating that there was no evidence of genome segment reassortment between viruses assigned to different genogroups. In contrast, comparing the L, N, and Gn/Gc trees, incongruities in the branch pattern within the clades was suggestive of genome segment reassortment within several genogroups (i.e., NSD, Sakhalin, Kasokero, Keterah, and Hughes). However, the number of viruses represented was insufficient to allow a statistically valid assessment.

### Structural analysis of proteins encoded in the L, S, and M segments.

#### L segment.

We generated an alignment of the L protein sequences for the 31 nairoviruses in our data set. Each of the functional domains and conserved motifs characteristic of bunyavirus L proteins were evident including region 1 (possible cap-snatching endonuclease), region 2 (unknown function), region 3 (polymerase module), and region 4 (capped-primer cleavage site and 5'vRNA binding site).^1^,^40^,^41^ In addition, the ovarian tumor (OTU)-like domain that is unique to nairoviruses among the *Bunyaviridae*, but also occurs in arenavirus L proteins, was found to be present in all nairoviruses except those detected in insects and spiders (Supplemental Figure 1). As reported previously,^13^ the L protein of tick-borne SBV, which is longest known among nairoviruses, may also lack an OTU-like domain. However, we did detect a sequence in the N-terminal region of SBV L that, although divergent, does show some homology with OTU-like domains of other nairoviruses (Supplemental Figure 1).

#### S segment.

We also conducted an alignment of the N protein sequences of the 31 viruses in our data set. The nucleoproteins were observed to be generally similar in size (479–516 aa) with the exception of Shayang spider virus 1 (SSV1) (583 aa) which has a long N-terminal extension, SBV (547 aa) which has a short C-terminal extension and Thiafora genogroup viruses (630 and 673 aa) which have long C-terminal extensions (Supplemental Figure 2). The C-terminal endonuclease domain displayed highest homology; lowest homology was in the central stalk domain. Significantly, 10 residues located in pockets that have been implicated in RNA or DNA binding in CCHFV,^42^,^43^ were universally conserved (identical or conservative substitutions) among the N proteins in this data set (Supplemental Figure 2). As observed previously, the caspase-3 cleavage site identified in the stalk domain of CCHFV (DEVD) and HAZV (DQVD)^44^,^45^ was also present in Thaifora genogroup viruses (DVLD and DILD) but a suitable motif does not appear to be present in other NSD genogroup viruses or any other viruses in the data set.

Alignment of the N protein sequences of the 27 viruses assigned to the nine genogroups indicated aa sequence identity (p-distance) was > 52% between viruses within genogroups and < 52% for viruses in different genogroups (Supplemental Table 1). The only exception was in the Tamdy genogroup in which amino sequence identity between HTV1 and WTV was estimated to be 47.7%. The analysis supported the assignment of NSD and CCHFV serogroup viruses as a single genogroup and supported the assignment of SAPV to the DGK genogroup.

#### M segment.

Alignment of the 30 available M segment polyglycoprotein sequences (there is no published M segment sequence for SBV) indicated that the sequence of Xinzhou spider virus (XSV) is incomplete and the sequences of other insect-associated nairo-like viruses (SSV1 and Shuangao bedbug virus 1 [SBV1]) are too distant for a useful alignment. We therefore analyzed the deduced aa sequences of long ORFs encoded in the M RNA segment of all 27 nairoviruses in the nine genogroups. In CCHFV, the M segment features an N-terminal signal peptide and multiple membrane-spanning domains, and is processed co-translationally by signal peptidases to generate a 140-kDa PreGn protein, an 85-kDa PreGc protein, and a double-membrane-spanning NSm protein. Pre-Gn and Pre-Gc are subsequently processed post-translationally by furin-like or subtilisin kexin isozyme-1 (SKI-1) proteases to generate: a mucin-like protein containing a large number of predicted O-glycosylation sites; a protein of unknown function designated GP38; virion envelope glycoprotein Gn; and virion envelope glycoprotein Gc.^1^,^46^

**Figure 3.**
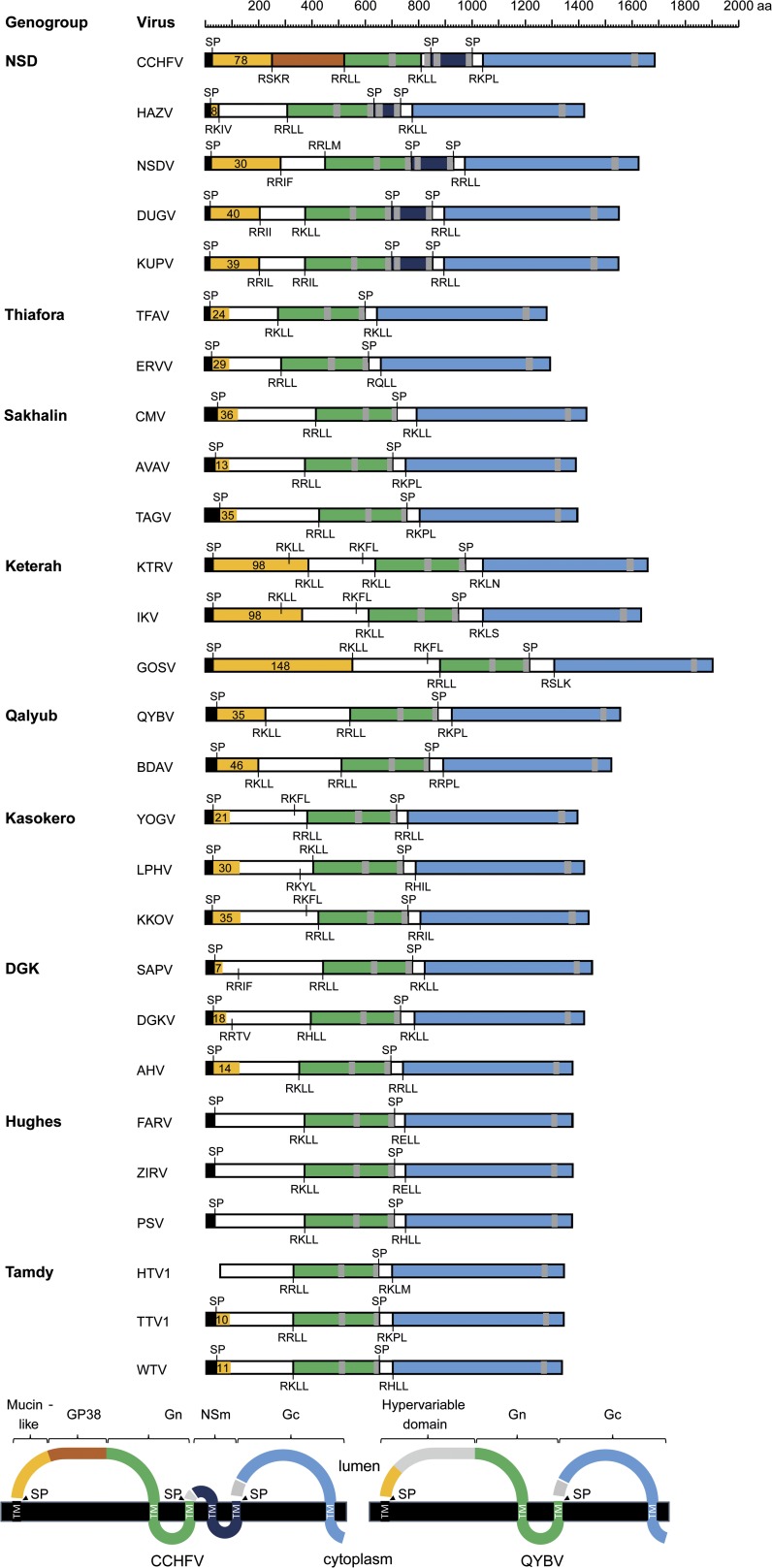
Schematic illustration of the structures and predicted membrane topologies of the polyglycoproteins encoded in the M segments of 27 nairoviruses. Regions corresponding to the mucin-like domain (orange), GP38 (rust), Gn (green), NSm (dark blue), and Gc (sky blue) are shaded. Predicted signal peptidase cleavage sites (SP) and potential furin-like and SKI-I cleavage sites are shown. The number of predicted O-linked glycosylation sites in the mucin-like domain are also shown.

**Figure 4.**
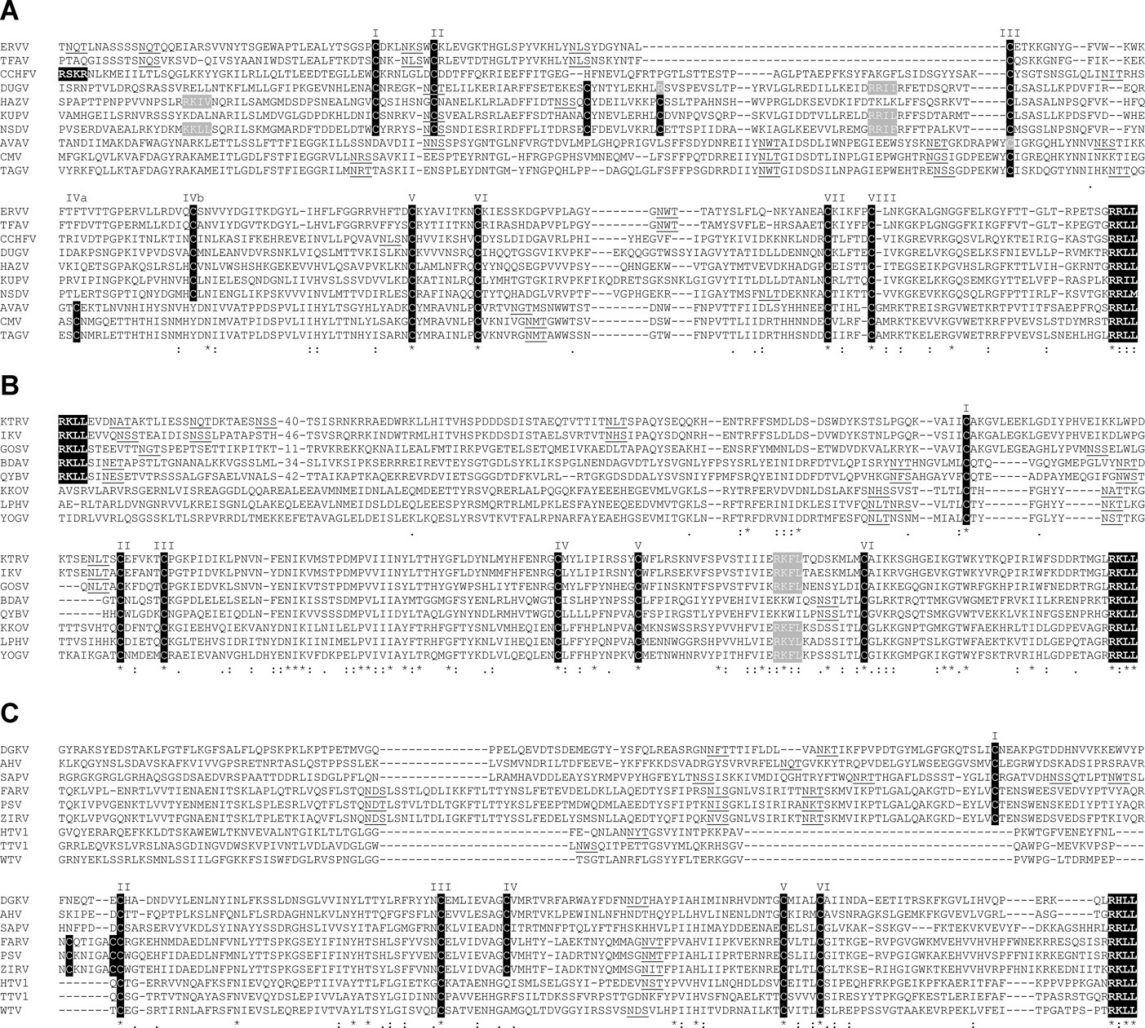
ClustalX alignments of the deduced amino acid sequences of the GP38-like domains of 27 nairoviruses. The alignments have been adjusted visually to emphasize alignment of closely positioned cysteine residues. (**A**) Viruses assigned to the Thaifora, NSD, and Sakhalin genogroups. (**B**) Viruses assigned to the Keterah, Qalyub, and Kasokero genogroups. (**C**) Viruses assigned to the DGK, Hughes, and Tamdy genogroups. Conserved cysteine residues are shaded in black and have been assigned roman numerals according to the order in which they appear in the sequence; the numerals do not necessarily correspond between the sets of sequences. An arginine residue (R) in the DUGV sequence (shaded in light gray) occurs at location at which a cysteine residue (C) is expected. A cysteine residue (C) in the AVAV sequence (shaded in light gray) was present as the minor proportion (8%) of ∼1,100 sequence reads, the remainder encoding a tyrosine residue (Y) as the result of a single transition mutation (guanosine to adenosine). Predicted SKI-1 cleavage sites (R[R/K/H][L/I][L/M]) are shown in bold face and shaded in black as is the furin cleavage site (RSKR) in CCHFV. Other potential SKI-1 cleavage sites are shaded in light gray. Predicted N-linked glycosylation sites are underlined. Identical (*), strongly conserved (:), and weakly conserved (.) residues as assigned in the Gronnet Pam250 matrix are indicated below the alignment. NSD = Nairobi sheep disease; DGK = Dera Ghazi Khan; DUGV = Dugbe virus; CCHFV = Crimean-Congo hemorrhagic fever virus.

Alignment of the polyglycoprotein sequences of the 27 nairoviruses indicated differences in total length (1,281–1,909 aa) due to structural variations that are largely genogroup-specific (Figure 3

). Firstly, the double-membrane spanning NSm protein located between the Gn and Gc proteins is present in all members of the NSD genogroup but absent from viruses assigned to other genogroups. Secondly, the N-terminal mucin-like domain varies significantly in length and in the number of predicted O-glycosylation sites; for example, it is most extensive in viruses assigned to the Keterah genogroup (98–148 predicted O-linked glycosylation sites), relatively small in viruses assigned to the DGK and Tamdy genogroups (7–18 predicted O-linked glycosylation sites), and totally absent from viruses assigned to the Hughes genogroup (Figure 3). Thirdly, the furin-like protease cleavage site (RSKR) that generates the mucin-like protein and GP38 in CCHFV was not conserved in any other viruses. However, an SKI-1 protease cleavage site (RKLL) was conserved at a similar locus in all viruses in the Keterah and Qalyub genogroups. Finally, although homology is generally quite low in the GP38 domain, and evident primarily between viruses within genogroups, patterns of conserved cysteine residues were observed to be genogroup-specific (Figure 4

). The NSD and Thiafora genogroups feature eight conserved cysteine residues (C_I_–C_VIII_); six of these appear to be conserved in the Sakhalin genogroup although one cysteine residue (designated C_IVa_) appears to be displaced laterally in the alignment; a subset of viruses in the NSD genogroup features two additional cysteine residues (Figure 4A). The Keterah, Qalyub, and Kasokero genogroups share six conserved cysteine residues (Figure 4B). The DGK and Hughes genogroups also share six conserved cysteine residues, four of which (C_II_, C_III_, C_V,_ and C_VI_) are also conserved in the Tamdy genogroup; the Hughes genogroup has an additional pair of cysteine residues (Figure 4C).

In contrast, the Gn and Gc glycoproteins are relatively well conserved in size and structural characteristics. All Gn glycoproteins are type 1 double-membrane-spanning proteins with an N-terminal ectodomain featuring 12 conserved cysteine residues and an endodomain loop featuring two consecutive zinc finger domains (ZFD I and ZFD II) (Supplemental Figure 3). Each has a single conserved N-glycosylation site immediately following the second cysteine residue and up to two other predicted N-glycosylation sites which occur at various locations that are not widely conserved. The Gc glycoproteins are each class I single-membrane-spanning proteins with two to four predicted N-glycosylation sites, the locations of which were largely genogroup-specific (Supplemental Figure 4). The Gc glycoproteins contain 28 conserved cysteine residues, four of which lie upstream of a predicted SKI-1 proteolytic cleavage site that would clip a small fragment from the N-terminus of the protein. An additional pair of cysteine residues occurs in all viruses except those assigned to the Thiafora and Tamdy genogroups, suggesting these residues form a unique disulphide bridge. Apparently unpaired cysteine residues also occur in viruses assigned to the DGK, Thiafora, and NSD genogroups.

**Figure 5.**
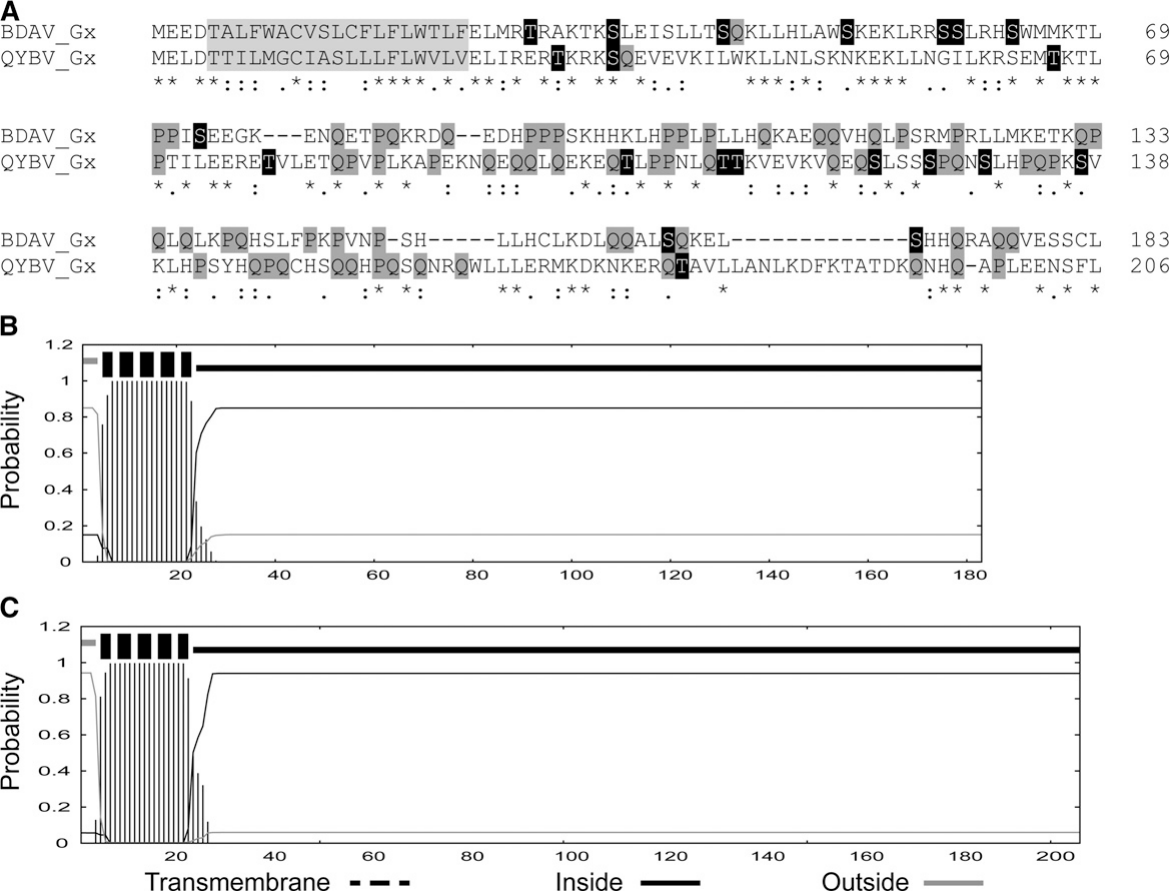
Structure of putative Gx proteins detected as alternative long ORFs in the M RNA segments of BDAV and QYBV (Qalyub genogroup). (**A**) Clustal X alignment of the Gx protein sequences. Predicted transmembrane domains are shaded in light gray. Predicted phosphorylation sites are shaded in black. Proline (P) and glutamine (Q) residues are shaded in dark gray. Identical (*), strongly conserved (:), and weakly conserved (.) residues as assigned in the Gronnet Pam250 matrix are indicated below the alignment. B. Predicted membrane topology of the BDAV Gx protein. C. Predicted membrane topology of the QYBV Gx protein. Membrane topologies were determined by using the TMHMM server Version 2.0 (http://www.cbs.dtu.dk/services/TMHMM-2.0). BDAV = Bandia virus; ORF = open reading frame; QYBV = Qalyub virus.

We also observed that the 5'-terminal region (in mRNA sense) of the M segments of the two Qalyub genogroup viruses contain alternative long ORFs encoding polypeptides of 206 aa (QYBV) and 183 aa (BDAV). Each commences 13 nucleotides downstream of the M polyprotein initiation codon and each is in favourable Kozak context for translation. These putative proteins (assigned as Gx proteins) share significant aa sequence homology (65.4% identity) and are predicted to be type I transmembrane proteins, each with a short acidic N-terminal ectodomain (luminal) followed by a hydrophobic transmembrane domain (residues 5–23) and a long hydrophilic endodomain (cytosolic) that is unusually rich in glutamine (Q) and proline (P) residues (totally 17.0% and 18.6% for QYBV and BDAV, respectively) (Figure 5

). The C-terminal domain was also predicted to be highly phosphorylated (NetPhos 2.0 server; http://www.cbs.dtu.dk/services/NetPhos). Similar long ORFs were not detected in the M segments of nairoviruses assigned to other genogroups.

### Vector and host associations.

We then analyzed the sources of isolation of the viruses assigned to each of the genogroups to identify patterns of association with hosts and vectors (Table 1 and Figure 1B). Viruses assigned to the Keterah, Kasokero, Qalyub, DGK, and Hughes genogroups were associated primarily with soft ticks (family Argasidae) whereas viruses assigned to the Tamdy, Sakhalin, and NSD genogroups were associated only with hard ticks (family Ixodidae). Although DGKV (DGK genogroup) has been reported to have been isolated from hard ticks (*Hyalomma dromedarii*), other viruses assigned to this genogroup (AHV, AMV, and SAPV) have been isolated only from soft ticks (*Argus* spp.). The vectors of viruses assigned to the Thiafora genogroup (TFAV and ERVV) are currently unknown.

Some patterns of association of genogroups with vertebrate hosts were also evident. As reported previously, Keterah genogroup viruses have a strong association with bats of the suborder Yangochiroptera, Kasokero genogroup viruses are associated with bats of the suborder Yingochiroptera and Thiafora genogroup viruses have been isolated only from shrews.^47^ Qalyub genogroup viruses were each isolated from rodents, ticks feeding on rodents, or ticks collected from their nests. The Hughes genogroup and Sakhalin genogroup each has a strong association with sea birds (gulls, murre, gannets, and cormorants). Viruses assigned to the DGK serogroup have been isolated primarily from birds with terrestrial habitats (pigeons, doves, and swallows); the exception is DGKV, which was isolated from a tick feeding on a camel. The Tamdy and NSD genogroups appear to be associated with domestic ungulates (camels, cattle, sheep, and goats) but the ecology of the hard tick species from which they have been isolated suggests that small mammals may also be involved in the ecology of these viruses.

## Discussion

Nairoviruses constitute a large group of tick-borne viruses that includes important pathogens of humans and livestock. As an emerging human pathogen with a high case-fatality rate, CCHFV has been the primary focus of detailed molecular and biological characterization to date.^1^ Although some other nairoviruses have also been the subject of investigation, a global view of nairoviruses, their relationships, biology, and pathogenic potential has been lacking. Here, we have conducted a comparative analysis of the genomic relationships between nairoviruses, facilitated by the availability of the complete coding sequences of 31 viral genomes, six of which we reported recently^47^ and 11 of which are reported here for the first time. Through this analysis, we identify nine genogroups comprising viruses with distinctive structural characteristics and ecological associations.

The assignment of viruses to the genus *Nairovirus* is currently based on antigenic cross-reactions detected using hemagglitination-inhibition, neutralization and immunoprecipitation tests, and assignment to nine serogroups (CCHF, NSD, DGK, Qalyub, Hughes, Sakhalin, Thiafora, Kasokero, and Keterah) is based on cross-reactions in indirect immunofluourescence and CF tests.^1^,^21^,^26^,^47^,^48^ All viruses assigned to a serogroup are assigned to a single nairovirus species, seven of which have been approved to date by the International Committee on Taxonomy of Viruses.^11^ Our phylogenetic analyses using complete L, M, and S segments allowed the assignment of 27 viruses to nine well-supported genogroups and 11 other nairoviruses were assigned to these genogroups by using the partial L gene sequences available from previous studies. The nine genogroups corresponded broadly to serogroups but with some important variations. Firstly, the NSD genogroup comprises viruses that have been assigned previously to the CCHF and NSD serogroups.^26^ In phylogenetic analyses, these viruses did not cluster consistently into CCHF-related (CCHFV and HAZV) and NSD-related (NSDV, KUPV, and DUGV) groups (Figures 1 and 2). This was supported by analysis of sequence identity in N proteins which were moderately high (57.4–75.7%) and displayed no serogroup-related patterns of homology (Supplemental Table 1). Each of these viruses has a similar genome organization, uniquely containing sequences in the M segment encoding the double-membrane-spanning NSm protein (Figure 3). We therefore consider that they should be regarded as a single genogroup (NSD). Secondly, although SAPV has previously been assigned to the Hughes serogroup,^17^ phylogenetic trees inferred from sequences encoded in the L, M, and S segments each indicate that it falls within the DGK genogroup (Figures 1 and 2). Analysis of N protein aa sequence homologies also indicated that SAPV is more closely related to the DGK serogroup (53.7–54.5% identity) than to the Hughes serogroup (40.3–41.9% identity) (Supplemental Table 1). This is also supported by the arrangement of cysteine residues in the “GP38-like” domain of the M segment polyglycoprotein in which DGKV, AHV, and SAPV each share six conserved cysteine residues with Hughes genogroup viruses (FARV, PSV, and ZIRV) but lack an additional pair of cysteine residues that are conserved only within the Hughes genogroup (Figure 4). We therefore assign SAPV to the DGK genogroup. Thirdly, we have identified that three viral genome sequences detected in ticks from China (HTV1, TTV1, and WTV)^12^ cluster phylogenetically with TDYV and BURV, which have previously been reported to be related genetically^38^,^49^ (Figures 1 and 2). Recognizing that HTV1, TTV1, and WTV are yet to be isolated and are represented only by genome sequences, we assign these putative viruses to the Tamdy genogroup.

The assignment of virus species in the family *Bunyaviridae* is currently based primarily on serological relationships, supported by considerations of virus ecology and genetic distance as assessed by aa sequence identity.^11^ Typically, bunyavirus species comprise viruses that cross-react strongly in neutralization tests and for which nucleoprotein or glycoprotein sequences differ by less than 7–10%. However, these criteria are relatively loosely defined, vary somewhat between genera and may be confounded by the potential for genome segment reassortment which appears to occur commonly among closely related bunyaviruses.^50^ Our data suggest that genogroups (reflecting evolutionary relationships) rather than serogroups (based on phenotypic relationships) may be a more useful basis of classification, at least for nairoviruses. Genogroup assignments, when adequately supported by bootstrap resampling, were consistent phylogenetically across all three segments and reflected similarities in other structural characteristics of the genome. Serogroup assignments, while having provided an invaluable basis for virus identification and classification for many years, are limited to the detection of cross-reactive epitopes which may be defined by few shared aa and not always reflective of the broader relationships or evolutionary history of viruses. The assignment of species within genogroups remains a more difficult consideration. As reported previously in a more limited analysis,^51^ aa sequence identity between the nairovirus nucleoproteins is highly variable but generally the level of divergence within genogroups (up to 52.3%) is far greater than the 7–10% divergence typical of bunyavirus species in some other genera. This may argue for the assignment of species within genogroups. However, we cannot exclude the possibility that genome segment reassortment contributes significantly to the ecological dynamics and evolution of nairoviruses. There has been no previous evidence of genome segment reassortment between different nairoviruses,^50^ and our data provide support for the view that reassortment, as reflected in different evolutionary histories of the L, M, and S segments, does not occur commonly between viruses assigned to different genogroups. Nevertheless, reassortment has been observed between CCHFV isolates from distant geographic locations^52^ and our analysis was suggestive of reassortment between viruses within genogroups. Consequently, we can see no reliable basis at this stage for the assignment of individual species below the genogroup level.

Our detailed analysis of deduced aa sequences focused primarily on the M segment which is the most variable in sequence, structure, and expression strategy. In particular, the hypervariable N-terminal region of the nairovirus polyglycoprotein displays characteristics that are generally reflective of genogroup assignments. Large variations in the length and potential for O-glycosylation of the “mucin-like” domain were particularly striking with viruses in some genogroups displaying few or indeed no predicted O-glycosylation sites. The function of the CCHFV mucin-like domain is presently not known but in Ebola virus a mucin-like domain in virion glycoprotein GP1 has been shown to be involved in multiple functions during infection and may have an important role in pathogenesis.^53^–^55^ Virion glycoprotein Gc of herpes simplex virus also contains a mucin-like domain that appears to be involved in cell entry and release of virus from infected cells.^56^ The influence of genogroup-associated variations in the length and extent of O-glycosylation in the mucin-like domain of various nairoviruses requires further study.

The GP38 domain of CCHFV is also of unknown function. Here we demonstrate that the structure of this domain varies among viruses assigned to different genogroups, as reflected in different patterns of conserved cysteine residues that are likely to be involved in the formation of intra- or inter-molecular disulphide bridges. All nairoviruses appear to share a common SKI-1 cleavage site (R[R/K/H][L/I][L/M]) at the C-terminus of this domain and, in CCHFV, this releases GP38 from the mature Gn glycoprotein.^46^ However, the furin-like cleavage site (RSKR) that releases CCHFV GP38 from the N-terminal mucin-like domain^46^ appears to be absent from all other nairoviruses. SKI-1 cleavage sites conserved at a similar location in viruses assigned to the Keterah and Qalyub genogroups may serve this function but it is unclear if the GP38-like domain could exist as a discrete protein in other nairoviruses. This is particularly intriguing for viruses in the Kasokero genogroup which share the same arrangement of cysteine residues with viruses in the Keterah and Qalyub genogroups but lack the relevant SKI-1 cleavage site and have a truncated mucin-like domain.

Of particular interest was the detection of unique alternative long ORFs in the M segments of Qalyub genogroup viruses. The proteins encoded in these ORFs are highly likely to be expressed in infected cells due to: 1) the proximity of each ORF to the 5'-terminus of the M mRNA; 2) the favourability of the Kozak contexts of the putative initiation codons; 3) conservation of the ORF in both QYBV and BDAV; and 4) the common distinguishing feature of a transmembrane domain. The function of these proteins (designated Gx) clearly require experimental investigation but their shared structural characteristics of a short N-terminal ectodomain, transmembrane domain, and long, highly phosphorylated endodomain resemble those of class IA viroporins.^57^ Viroporins have been identified in a wide range of animal viruses, forming oligomers that insert into cellular membranes, disrupting cellular physiology.^57^–^62^ Several viroporins have been found to be crucial for viral pathogenicity.^57^,^63^,^64^

Ecological associations of nairovirus serogroups with specific categories of hosts and tick vectors have been observed previously.^47^,^51^ Indeed, similarities in the phylogenies of nairoviruses and their vectors have been suggested to be indicative of coevolution that dates to the divergence of hard ticks (Ixodidae) and soft ticks (Argasidae) 120–92 million years ago.^51^ Our data generally support this conclusion. Furthermore, the inclusion in phylogenetic analyses of nairovirus-like sequences derived from arachnids and insects indicates that the evolutionary origins of nairoviruses may be considerably deeper. Nevertheless, there are exceptions to this general trend of host and vector association (e.g., the isolation of DGKV from hard ticks feeding on a camel) and this is likely to be indicative of a more complex ecology in which host switching plays a role in the epidemiology and evolution of nairoviruses.

## Supplementary Material

Supplemental Datas.

## Figures and Tables

**Table 1:** Viruses and sequences used in this study

Virus	Strain	Approved species	Genogroup	Date of isolation	Place of isolation	Species of isolation	Reference	Genbank
Crimean-Congo hemorrhagic fever (CCHFV)	IbAr10200	Crimean-Congo hemorrhagic fever virus	NSD	May 11, 1966	Sokoto, Nigeria	Hard ticks (*Hyalomma excavatum*) collected from a camel	65	NC_005300
								NC_005301
								NC_005303
Hazara (HAZV)	JC280	Not assigned	NSD	July 20, 1964	Gitidas, Hazara District, Pakistan	Hard ticks (*Ixodes redikorzevi*) collected from a vole (*Alticola roylei*)	66	KP406723
								KP406724
								KP406725
Nairobi sheep disease (NSDV)	G619	Not assigned	NSD	November 6, 1954	Bhanjanager, Ganjam District, Orissa, India	Hard ticks (*Haemaphysalis intermedia*) collected from goats	67	EU697949
								EU697950
								AF504294
Kupe (KUPV)	K611	Not assigned	NSD	October 1999	Nairobi, Kenya	Hard ticks (*Amblyomma gemma*) from cattle	68	EU257628
								EU257627
								EU257626
Dugbe (DUGV)	ArD44313	*Dugbe* virus	NSD	November 1985	Bouroufaye, Senegal	Hard ticks (*Amblyomma variegatum*)	69	NC_004159
								NC_004158
								NC_004157
Chim CHIMV)	LEIV-858Uz	*Qalyub* virus	Qalyub	July 10, 1971	Chim Village, Kashkadarynsk Region, Uzbekistan	Soft ticks (*Ornithodoros tartakovskyi*) collected from gerbil burrow	33	KF801656
Qalyub (QYBV)	ErAg370	*Qalyub* virus	Qalyub	August 28, 1952	Qalyub, Qalyubiya Province, Egypt	Soft ticks (*Ornithodorus erraticus*) from a rat nest	14	KU343160
								KU343161
								KU343162
Bandia (BDAV)	IPD/A611	*Qalyub* virus	Qalyub	February 26, 1965	Bandia Forest, Thies Region, Senegal	Rodent (*Mastomys* sp.) (also from ticks [*O. erraticus*] collected from rodent burrow)	15	KU343148
								KU343149
								KU343150
Geran (GERV)	LEIV-10899Az	*Qalyub* virus	Qalyub	September 11, 1985	Goranboy District, Azerbaijan	Soft ticks (*Ornithodoros verrucosus*) collected from gerbil (*Meriones erythrourus*) burrow	36	KF801649
Farallon (FARV)	CalAr846	*Hughes* virus	Hughes	July 1965	South Farallon Island, California, USA	Soft ticks (*Ornithodorus* sp.) from seagull nest	20	KU343154
								KU343155
								KU343156
Punta Salinas (PSV)	CalAr888	*Hughes* virus	Hughes	October 14, 1967	Punta Salinas, Huacho, Peru	Soft ticks (*Ornithodorus amblus*) from guano of bird colony	19	KU343157
								KU343158
								KU343159
Zirqa (ZIRV)	A2070-1	*Hughes* virus	Hughes	November 2, 1969	Zirqa Island, Abu Dhabi, Persian Gulf	Soft ticks (*Ornithodorus muesebecki*) from cormorant nests	18	KU343169
								KU343170
								KU343717
Caspiy (CASV)	LEIV-63Az	*Hughes* virus	Hughes	1970	Gil Island, Caspian Sea, Azerbaijan	Seagull (*Larus argentatus*)	35	KF801658
Raza (RAZAV)	829	*Hughes* virus	Hughes	1962	Raza Island, Gulf of Mexico, Mexico	Soft ticks (*Carios denmarki*)		AY359529
Avalon (AVAV)	CanAr173	*Sakhalin* virus	Sakhalin	July 31, 1972	Great island, Newfoundland, Canada	Hard ticks (*Ixodes uriae*) from Herring gull (*Larus argentatus*)	22	KU343145
								KU343146
								KU343147
Clo Mor (CMV)	ScotAr7	*Sakhalin* virus	Sakhalin	June 15, 1973	Clo Mor, Cape Wrath, Scotland	Hard ticks (*Ixodes uriae*) in nesting sites for Murre sea birds (*Uria aalge*)	22	KU343139
								KU343140
								KU343141
Tillamook (TILV)	RML86	*Sakhalin* virus	Sakhalin	1970	Oregon, USA	Hard ticks (*Ixodes uriae*)		AY359530
Sakhalin (SAKV)	LEIV-71C	*Sakhalin* virus	Sakhalin	August 18, 1969	Tyuleniy Island, Sea of Okhotsk, USSR	Hard ticks (*I. uriae*) in nesting sites for Murre sea birds (*U. aalge*)	34	KF801659
Paramushir (PMRV)	LEIV-2268Ku		Sakhalin	September 2, 1972	Paramushir Island, Sea of Okhotsk, Russia	Hard ticks (*Ixodes signatus*) from gannet (*Phalacrocorax pelagicus*) nest	34	KF801657
Taggert (TAGV)	MI14850	*Sakhalin* virus	Sakhalin	January 1, 1972	Macquarie Island, Southern Ocean, Australia	Hard ticks (*I. uriae*) from seabird rookery	23	KU343166
								KU343167
								KU343168
Dera Ghazi Khan (DGKV)	JD254	*Dera Ghazi Khan* virus	Dera Ghazi Khan	April 4,1966	Dera Ghazi Khan District, Pakistan	Hard ticks (*Hyalomma dromedarii*) collected from a camel	24	KU343151
								KU343152
								KU343153
Abu Hammad (AHV)	EgArt1194	*Dera Ghazi Khan* virus	Dera Ghazi Khan	June 7, 1971	Abu Hammad Sharqiya, Egypt	Soft ticks (*Argas hermanni*) collected from a pigeon house	25	KU343142
								KU343143
								KU343144
Abu Mina (AMV)	EgArt4996	*Dera Ghazi Khan* virus	Dera Ghazi Khan	1963	Bahig, Egypt	Dove and subsequently soft ticks (*Argas streptopelia*)	25	AY357716
Sapphire II (SAPV)	52301-14	*Hughes* virus	Hughes	July 28, 1969	Sunday Canyon, Randall County, Texas, USA	Soft ticks (*Argas cooleyi*) from swallows (*Petrochelidon pyrrhonota*)	17	KU343163
								KU343164
								KU343165
Ahun (AHUNV)	B8 (sequence only)		Not assigned	2009	France	Bat (*Myotis mystacinus*)	70	KF170224
Tamdy (TDYV)	LEIV-1308Uz	Not assigned	Tamdy	August 1971	Tamdy District, Uzbekistan	Hard ticks (*Hyalomma asiaticum*) collected from sheep	49	KF801653
Burana (BURV)	LEIV-Krg760	Not assigned	Tamdy	April 1971	Burana, Kirgizia, Kyrgyzstan	Hard ticks (*Haemaphysalis punctate*) collected from cattle	38	KF801651
Huangpi tick virus 1 (HTV1)	H124-1 (sequence only)	Not assigned	Tamdy	2013	Huangpi, Hubei, China	Hard ticks (*Haemaphysalis doenitzi*)	12	KM817667
								KM817706
								KM817734
Tacheng tick virus 1 (TTV1)	TC253 (sequence only)	Not assigned	Tamdy	2012	Tacheng, Xinjiang, China	Hard ticks (*Dermacentor marginatus*)	12	KM817683
								KM817717
								KM817743
Wenzhou tick virus (WTV)	TS1-2	Not assigned	Tamdy	2012	Wenzhou, Zhejiang, China	Hard ticks (*Haemaphysalis hystricis*)	12	KM817685
								KM817718
								KM817745
South Bay (SBV)	SBV-H1 (sequence only)		Not assigned	April 2013	Suffork County, New York, USA	Hard ticks (*Ixodes scapularis*)	13	KJ746877
								KJ746878
Artashat (ARTSV)	LEIV-2366Az		Not assigned	1972	Armenia	Soft ticks (*Ornithodoros alactagalis*) from five-toed jerboa (*Allactaga elater*) burrow	71	KF801650
Sanxia water strider virus 1 (SWSV1)	SXSSP08		Not assigned	2012	Sanxia, Hubei, China	Water strider (unidentified Gerridae)	12	KM817674
								KM817711
								KM817737
Xinzhou spider virus (XSV)	XZZZ-2		Not assigned	2013	Xinzhou, Shanxi, China	Spider (*Neoscona nautica*)	12	KM817702
								KM817729
								KM817762
Thiafora (TFAV)	AnD 11411	*Thiafora* virus	Thiafora	February 17, 1971	Bandia, Senegal	Shrew (*Crocidura* sp.)	48	KR537450
								KR537451
								KR537452
Erve (ERVV)	Brest/An221	*Thiafora* virus	Thiafora	May 5, 1982	Saulges, Mayenne, France	White-toed shrew (*Crocidura russula*)	72	JF911697 JF911698
								JF911699
Keterah (KTRV)	P61361	Not assigned	Keterah	February 11, 1966	Keterah, Kelantan, Malaysia	Soft ticks (*Argae pusillus*) from lesser Asian yellow house bat (*Scotophilus kuhlii* [*temmincki]*)	73	KR537447
								KR537448
								KR537449
Uzun Agach (UAV)	LEIV-Kaz155	Not assigned	Keterah	1977	Alma-Ata District, Kazakhstan	Bat (*Myotis blythii*)	74	KJ744032
Issyk-Kul (IKV)	LEIV-315K	Not assigned	Keterah	May 15, 1970	Dzety Oguzsk	Common noctule bat (*Nyctalus noctula*)	75	KR537441
					Region, Kyrgyzstan			KR537442
								KR537443
Gossas (GOSV)	DakAnD 401	Not assigned	Keterah	November 19, 1964	Gossas, Senegal	Bat (*Tadarida sp.*)	47	KR534876
								KR534877
								KR534878
Yogue (YOGV)	DakAnD 56	*Kasokero*	Yogue	June 19, 1968	Bandia, Senegal	Egyptian fruit bat (*Rousettus aegyptiacus*)	48	KR537453
								KR537454
								KR537455
Kasokero (KKOV)	Z-52963 and Z-52969	Not assigned	Yogue	August XX, 1977	Masaka District, Uganda	Egyptian fruit bat (*Rousettus aegyptiacus*)	4	KR537444
								KR537445
								KR537446
Leopards Hill (LPHV)	11SB17	Not assigned	Yogue	November 29, 2011	Lusaka, Zambia	Leaf-nosed bats (*Hipposideros gigas*)	76	NC_025831
								NC_025832
								NC_025833
