# Supplementary material for: A Global Genomic Characterization of Nairoviruses Identifies Nine Discrete Genogroups with Distinctive Structural Characteristics and Host-Vector Associations
Source: Am J Trop Med Hyg. 2016 May 4;94(5):1107–22. doi: 10.4269/ajtmh.15-0917 (PMC4856612; doi:10.4269/ajtmh.15-0917)
Supplement: Supplementary file 1 [file SD7.pdf]

```
AVAV     DGNCLYH-ALACGIVENQQPDSYKTVKEIVKESAGLFWKCTAEAQVSGEELSD------------------------------YLIRISKP
CMV      DGNCLYH-ALACGLVEEQQPDSYKTVKELVKESAGLFWNNTAEAQVSEEDLAS------------------------------YLVRISRP
TAGV     DGNCLYH-SLACGMIEEQQPDSYKLIKEQVREAAGLFWDTTEETKTTGEDLNG------------------------------YLARIMKP
CCHFV    DGNCFYH-SIAELTMPNKTDHSYHYIKRLTESAARKYYQEEPEARLVGLSLED------------------------------YLKRMLSD
NSDV     DGNCFYH-SIAELFVPNKNDFSFRLVKQHLELAARRFFEEESEAKGLGLSLEK------------------------------YLEVAMCD
DUGV     DGNCFYH-SIAELFFDVKTPSSFRKVKEHLQLAAEVYYDTEPEAVGTGISKDE------------------------------YIKVAMKD
KUPV     DGNCFYH-SIAELFFDVKTPFSFRKVKEHLRLAADAFYDTEPEAIGTGVTKEE------------------------------YIQAAMKD
HAZV     DGNCFYH-SLAELYIPNKSDHAYRLVKNELREAAEKYFPTEPEAAATGMRLDE------------------------------YLDTALRD
ERVV     DGNCFYR-ALSRLHSESRTSNEHLYYRLLIPDAVDKYFDIEPEAIGLGLNKQE------------------------------YVSKAILD
TFAV     DGNCFYR-ALSRLHSKDKTSDEHLYYRLLIPDAAQRYFDTEQEAVGLGLTKEE------------------------------YASRAILD
KKOV     DGTCFFS-SVSKYIFN--TTEMWKTVKSTCANYARAHWQEVMEMDRRYQSPET------------------------------YIEDLMND
LPHV     DGTCFFS-SVSKYIFN--TTELWQTVKSTCANYARAHWKEVMEMDRQYAEADA------------------------------YISDLMRD
YOGV     DGTCFFS-SVSKYIFN--TTEQWRAVKTTCANYARANWDTVMEIDRHYQNADH------------------------------YVNDLMRD
IKV      DGRCFFR-AMAKFMFN--SSDEWLIVKRACIEYSRQHWNRFLVYTRLYPSSAD------------------------------YERDIMRD
KTRV     DGRCFFR-AMAKFMFN--SSDEWLIVKRACIEYARQHWNKFLVFTRLYQTPAD------------------------------YERDIMRD
GOSV     DGKCFFR-AMAKFMFN--SDEEWLLVKRACVEFARQHWDRFLEFTRNYQRAND------------------------------YERDIMRD
BDAV     DGNCFFR-AVSAFLYD--TQNGWNEVKSICREYAKAKWDTLIDLPTRYQNPDH------------------------------YCRDQLPD
QYBV     DGNCFFR-AVSTFLYD--TQNGWIEVKNMCREFAETNWDELPGVHQYFQDPEH------------------------------YARESKRE
HTV1     DGDCFFR-CAALHLLK--SEVEVSRMKNIILSYALNNWDSLPELREYYSESAE------------------------------YIRDFNSP
TTV1     DGDCFFH-CLAKQLP----EVSVSRLKGIITSYALRNWDTLTEAPRFYSDPKD------------------------------YERELNRA
WTV      DGDCFFY-CVSLHLSG--GSLKVDRIKGIITSFALRNWEDIYEARLFYGTPEA------------------------------YIADLYSP
DGKV     DGNCFFY-SVSFLLFE--SLSEWRSIKNTIASFAAANWGQCVQAKLNYANSSD------------------------------YRADMLRN
AHV      DGNCLFY-SLSYLMFD--TLGEWRSLKATISNFALTNWGQCNLAKLDYKSPQE------------------------------YVADLNRN
SAPV     DGNCFFY-AISYLLFG--NLKEWRAVKSTVERWSSANWTECREARHSYNSSAE------------------------------YRAALMQS
FARV     DGNCFFR-AFAYLFFD--TEEMWDTVKGTALGYARQHWSECHGAKGVYNYRAENEIKSEKALYSSVLRGNA-TENVTRRGLDLYLEDATKE
PSV      DGNCFFR-SFAYLFFD--SEEMWPAVKNTALGYARQHWAECYGARGLYENRAIEEQKTMRASYSAVVKGNV-QERSTERGKELYLEEASKD
ZIRV     DGNCFFR-AFSVLFFD--QEDQWRTVKNTAVSFAKKSWSECTGAKQQYVNKAEKEKAQHKLTYRDVTLGDTGLDMLSRRGREVYIEEAQKE
         ** *::                               :                                             *
SBV      RGGCQGNDRAVTLGWERFTLDPEGPVG--ISSMQLLSWIVTGEKDPKPMTVES------------------------------VITDAAL-
          * *                                 :

AVAV     NEWGSTLEVNFFAQKTKLTVYVWHEDANKHCDHVMRYGQDGVANAIN-ILHRKNHFDFL
CMV      NEWGSTLEVNFFSQKTKLTVYVWHEDVNKHCNYVLRYGNDRIIESIN-ILHRKNHFDYL
TAGV     NEWGSSLEVNFFSQKAKVTVYIWHEDASKHCDYVVRYGEDPMLESIN-IMHRRNHYDYL
CCHFV    NEWGSTLEASMLAKEMGITIIIWTVAASDEVEAGIKFGDGDVFTAVNLLHSGQTHFDAL
NSDV     NEWGGSLEASMLAKHLDITIVIWVIEGPSRVAAAVKFGPGDVAGAINLLHTGYNHFDAL
DUGV     NEWGGSLEASMLSKHLQTTIILWVVNSTEQVTAAIKFGPGRVSTALNLMHVGRTHFDAL
KUPV     NEWGGSLEASMLSKQLQITIILWVVNQTEQVTAAIKFGPGRVSTALNLMHVGRTHFDAL
HAZV     NEWGGSLEAAMLSRHLGLTVVIWLVDGSNRVVGATRFGKGSLKTALHLLHSGLTHFDAL
ERVV     GEWAGSLEASMLSKFLDITIIIWIVDDSGTIISANRYGEGRPSQAYNLCMVGNAHFDSL
TFAV     GEWAGSLEASMLSKFLDITIIIWIIDGSGTITSAQRYGNNKPSKAYNLCLIGNTHFDSL
KKOV     QYWGGSVEAEILSKALNMTIHIWVSGDGVWVNNARRWGADQIHASLNLIHVHGGHFNLL
LPHV     QYWGGSIEAEILSKALNMTIYIWVSGDGVWVSNARRWGGEPIQTSLNLIHVHGGHFNLL
YOGV     QYWGGSVEAEILNKALNMTVHIWVSDDGVWVQNARRWGREAVAASLNLVHVHGGHFNLL
IKV      DYWGGSLEAEVLSDLYNLTIHFWVTDDHQWIHHVQRWKNNAPHMSINLLFN-QNHFDLL
KTRV     DYWGGSLEAEVLSDLYNLTIHFWVTNDHQWIHHVQRWKNNAPHMSINLLFN-QNHFDLL
GOSV     DYWGGSLESDILSELYNVTIHFWVTDDHLWIHHVQRWGLEAPHISINLLFN-QNHFDLL
BDAV     GYWGGSVEAEILSKALNISIVFWRCDDDVWVTNACKWGQGDFRTSINLLHLRQDHFDYL
QYBV     GYWGGSVEAEILSKLLKLTVIFWKCEDDVWVTQGIRWGDGNYLTAINLLHIQFDHFDFL
HTV1     GYWGGSIEAEIINHAFGVPVVLWHTSDWTMTVAAKFWLRRHGPEPELNLVYQGSHFQYL
TTV1     GYWGGTTEAEIINHSFGVPVVIWTTEDKKLTSAVQVWTRKHGNLPELHLLHTGTHFMCL
WTV      GYWGGSVEAEILNKAYGMPIIIWSSTDGIYSTDVRIWTRKYDGMPEMNLIAAGRHFQYL
DGKV     YYWGGSVEAEILSKALNITIILWEADVSENVVTATKYGPGLVSTALN-LKLCQGHIEPL
AHV      YYWGGTIEAEIVSLALNTTVVLWCTDVLDNVVDAFKYGQEPVSRALN-LRLSNAHVSPL
SAPV     GYWGGSVEADILSRALNTTIILWTVDISGRVEIAYKYGKSNVSTSLN-LKLYKGHFNAL
FARV     GYWGGTDEAEMLASALNVTIVIWNVNTDMKVLDVQKFGTDSVPRAFN-IVRCGAHFDAL
PSV      GYWGGTDEAEMLAKALNLTIIIWNVNSDLKVLDVQKFGPHTVERAFN-IVKTGAHFDAL
ZIRV     GYWGGTNEAEMISKSLGVTIIIWEVNSDMKVVSVQKFGKDKVSGSFN-IMKLDAHFDGL
           *..  :  ..      .: .*             :       .         *   *
SBV      QWWPEIN-----LDCDTEVMVVADSMSDYL-SRKP-WEAQSVDLLTLALLCI--HLDKG
           *        .       : .              :                 *
```

Supplemental Figure 1. ClustalX alignment of the deduced amino acid sequences of the OTU-like protease domain in the L proteins of 27 nairoviruses assigned to nine genogroups as well as SBV. Identical (*), strongly conserved (:), and weakly conserved (.) residues as assigned in the Gronnet Pam250 matrix are indicated below the alignment. Highly conserved residues are shaded in gray. OUT = ovarian tumor.

Supplementary Figure 2

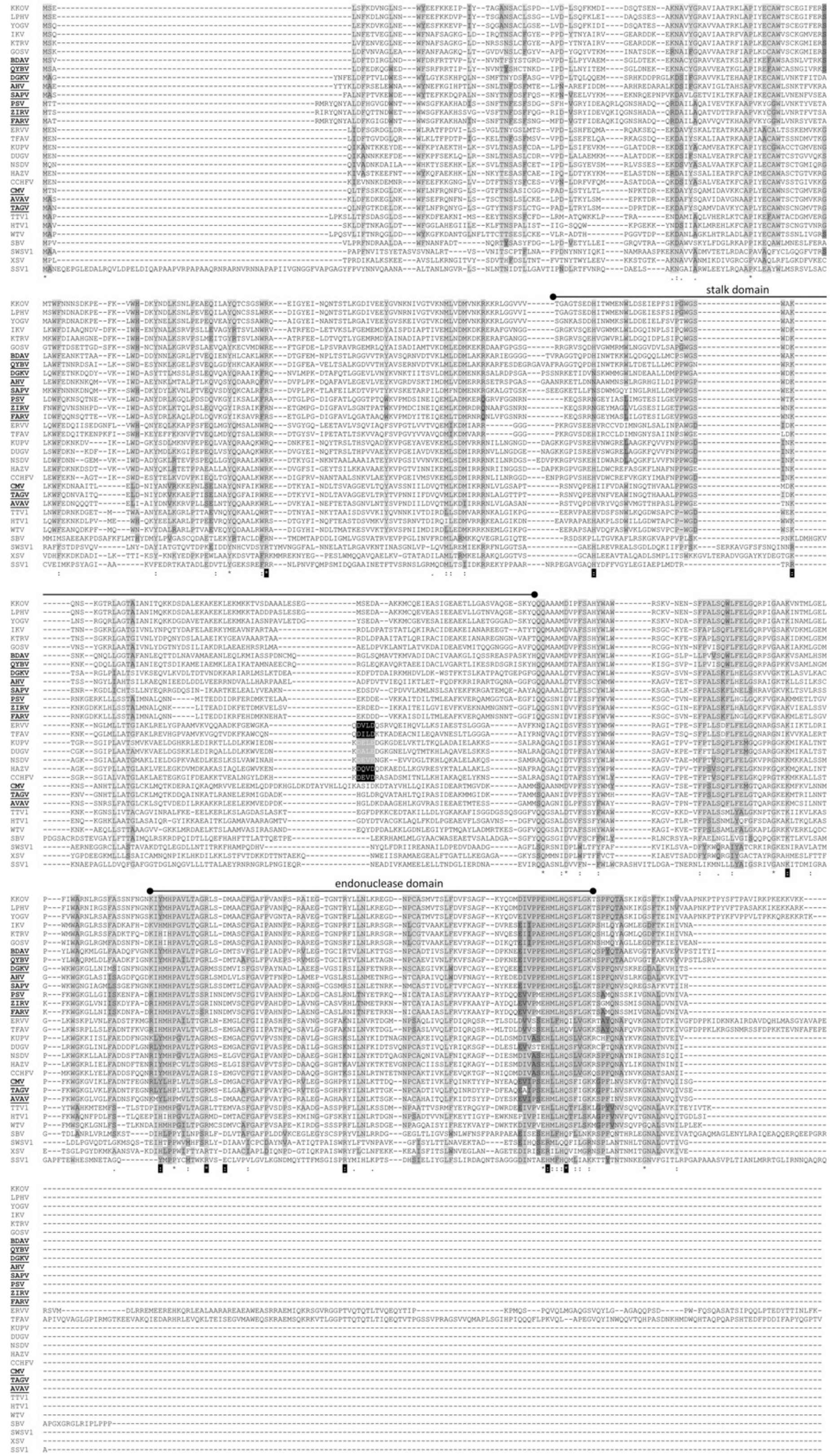

Supplemental Figure 2. ClustalX alignment of the deduced amino acid sequences of the nucleoproteins of 31 nairoviruses. Relatively conserved residues are shaded in gray. The stalk domain and endonuclease domains are marked and the caspase 3 cleavage site identified in CCHFV, HAZV, TFAV and ERVV is shown. Identical (*), strongly conserved (:), and weakly conserved (.) residues as assigned in the Gronnet Pam250 matrix are indicated below the alignment. Ten residues that have been implicated in DNA or RNA binding to the CCHFV N protein are identified by black shading of the Gronnet assignments. Viruses for which sequences were generated in this study are shown in bold and underlined. CCHFV = Crimean-Congo hemorrhagic fever virus; ERVV = Erve virus; HAZV = Hazara virus; TFAV= Thiafora virus.

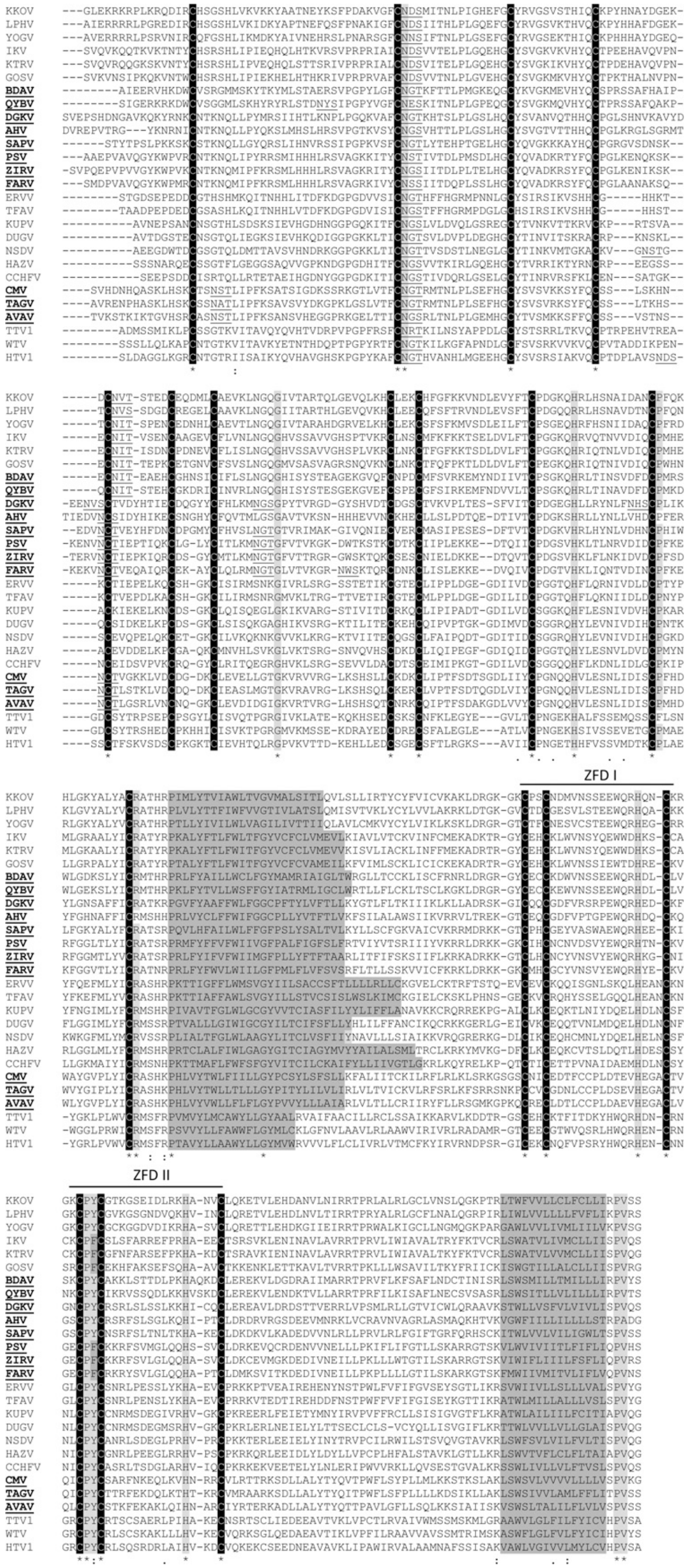

SUPPLEMENTAL FIGURE 3. ClustalX alignment of the deduced amino acid sequences of the Gn proteins of 27 nairoviruses. Conserved cysteine residues are shaded in black and two predicted transmembrane domains are shaded in dark gray. Predicted N-glycosylation sites are underlined. Two zinc finger domains (ZFDI and ZFDII) are indicated. Identical (*), strongly conserved (:), and weakly conserved (.) residues as assigned in the Gronnet Pam250 matrix are indicated below the alignment. Viruses for which sequences were generated in this study are shown in bold and underlined.

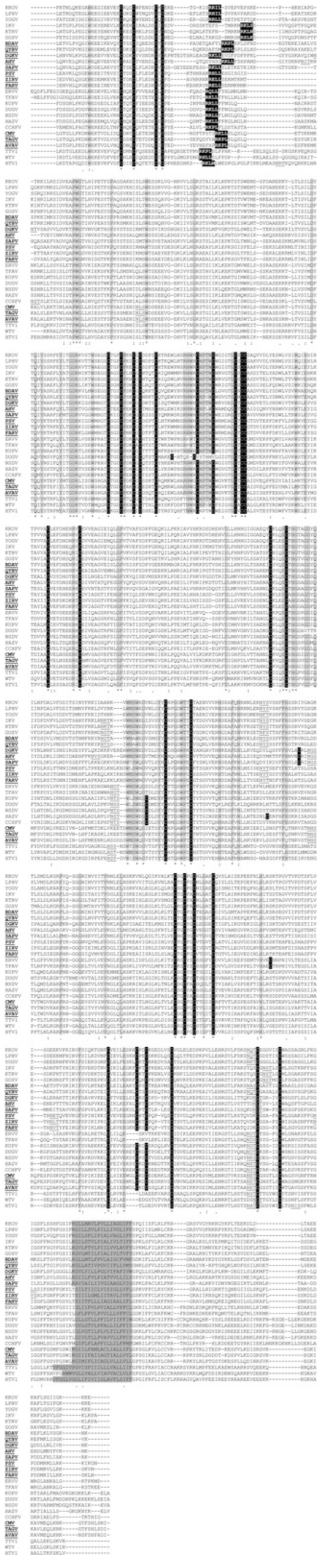

SUPPLEMENTAL FIGURE 4. ClustalX alignment of the deduced amino acid sequences of the Gc proteins of 27 nairoviruses. Conserved cysteine residues are shaded in black and one predicted transmembrane domain is shaded in dark gray. Potential SKI-1 proteolytic cleavage sites near the N-terminus are shaded in black and underlined. Predicted N-glycosylation sites are underlined. Identical (*), strongly conserved (:), and weakly conserved (.) residues as assigned in the Gronnet Pam250 matrix are indicated below the alignment. Universally conserved or very highly conserved residues are highlighted in gray.

Supplemental Table 1

Amino acid sequence identities (%) of nairovirus N proteins as determined by p-distance estimations in MEGA 6

Serogroup		CCHFV		NSD			Thiafora		Sakhalin			Keterah			Qalyub		Kasokero			DGK		Hughes				Unassigned		
Genogroup	Virus	CCHFV	HAZV	NSDV	DUGV	KUPV	TFAV	ERVV	TAGV	AVAV	CMV	KTRV	IKV	GOSV	QYBV	BDAV	KKOV	YOGV	LPHV	DGKV	AHV	SAPV	ZIRV	FARV	PSV	HTV1	TTV1	WTV
NSD	CCHFV	100																										
	HAZV	61.6	100																									
	NSDV	63.4	64.4	100																								
	DUGV	58.3	57.4	62.3	100																							
	KUPV	59.0	59.5	64.6	75.7	100																						
Thiafora	TFAV	46.5	43.5	47.2	45.8	43.4	100																					
	ERVV	45.6	43.5	45.4	45.8	44.0	72.0	100																				
Sakhalin	TAGV	44.7	44.7	44.4	43.3	43.3	40.3	41.2	100																			
	AVAV	44.2	43.6	42.4	44.0	42.4	39.8	38.9	66.0	100																		
	CMV	43.7	43.7	43.7	42.8	42.4	40.0	38.9	79.2	63.7	100																	
Keterah	KTRV	36.8	34.5	36.3	33.3	34.3	34.0	34.5	34.5	38.2	36.3	100																
	IKV	36.1	35.2	37.3	34.7	36.6	34.3	35.2	35.9	38.4	37.0	87.7	100															
	GOSV	35.9	34.7	36.1	33.6	34.0	33.3	32.4	35.4	38.0	37.0	68.3	65.3	100														
Qalyub	QYBV	38.7	36.6	38.7	36.6	35.2	34.3	31.5	36.6	37.7	38.0	41.2	43.1	43.7	100													
	BDAV	38.4	34.7	37.3	37.3	35.2	35.2	31.7	37.0	38.0	37.7	43.1	43.1	45.1	73.6	100												
Kasokero	KKOV	38.2	37.3	38.9	38.7	37.5	37.0	33.1	35.0	42.1	37.0	49.5	48.8	48.8	51.4	50.2	100											
	YOGV	37.7	35.2	38.9	38.0	38.7	37.0	32.6	35.0	39.4	36.8	45.5	49.8	50.2	49.8	51.6	81.0	100										
	LPHV	38.9	36.8	38.9	38.7	38.4	37.7	33.8	36.3	42.4	38.4	49.3	48.4	50.0	50.9	51.2	91.7	80.3	100									
DGK	DGKV	36.1	34.7	36.3	34.5	35.0	31.2	31.5	33.1	33.8	33.3	36.6	36.3	38.7	38.0	36.8	39.6	41.0	38.4	100								
	AHV	37.5	39.8	37.0	34.3	35.6	34.0	34.0	35.6	38.7	34.3	37.0	37.5	36.8	34.7	34.5	38.4	38.9	38.4	60.9	100							
	SAPV	36.8	35.0	33.3	33.1	33.8	33.3	30.8	33.6	35.4	33.6	36.1	37.7	36.6	37.3	38.7	39.6	40.0	39.8	54.5	53.7	100						
Hughes	ZIRV	33.3	33.8	33.1	33.3	33.8	32.2	32.9	32.2	34.5	31.5	34.7	35.2	34.0	31.2	31.7	33.3	33.3	33.3	45.6	43.1	41.9	100					
	FARV	32.6	32.9	32.2	32.6	32.6	31.5	32.2	30.3	32.2	30.6	32.9	33.6	34.3	31.2	31.9	32.9	33.8	33.1	44.2	45.4	41.9	76.2	100				
	PSV	33.1	33.8	32.6	33.6	34.3	31.9	32.4	31.2	32.9	31.9	33.8	34.0	35.0	31.7	33.1	32.2	33.1	32.6	43.3	44.4	40.3	73.8	86.6	100			
Tamdy	HTV1	36.1	35.2	36.8	35.4	36.6	34.0	32.9	38.0	37.7	37.7	30.6	29.6	32.2	34.3	32.9	35.0	35.0	34.7	32.2	33.1	33.8	31.0	30.6	30.6	100		
	TTV1	35.0	36.6	36.6	34.5	35.9	33.8	33.1	36.6	37.7	32.3	34.3	35.2	34.0	34.3	35.2	35.2	35.4	35.4	33.1	34.5	33.8	31.7	31.0	32.4	52.8	100	
	WTV	35.6	34.5	34.7	35.0	32.6	31.0	31.9	36.6	35.9	34.5	31.5	32.6	31.0	33.6	35.6	33.8	35.2	33.6	30.6	33.6	31.9	32.4	31.5	31.9	47.7	52.1	100
